# 

**Published:** 1913-03-01

**Authors:** 


### METROPOLITAN HOSPITAL SATURDAY FUND

Sir Savile Crossley, Bart., presided on Saturday evening at the first meeting of the re-constituted Board of Delegates of the Hospital Saturday Fund for 1913 held at 34 Red Lion Square, High Holborn. There was a large attendance. The certificates of delegates—264—which were duly verified, comprised 116 from contributing firms; 75 from local committees; and 73 from participating institutions. Sir Savile, who was unanimously re-elected Chairman of the Fund, said he was glad to say that he had been chairman for thirteen years, but he could only accept the position subject to the reservation that he would be going abroad at the end of March. He would, however, be back again by the end of June. He would not be able to continue after this year, as he would be taking up other work in the country. He felt it a great honour to be associated with the Fund, and thanked them for the honour they had done him in again electing him. Other hon. officers elected included Mr. Harry Gower (Chairman of the Council); the Hon. H. L. W. Lawson (Treasurer); Mr. B. Marwood (Sub-Treasurer); Mr. Gerald Carter (Hon. Standing Counsel); Mr. H. Owen (Hon. Solicitor); and Messrs. J. R. Dilley, D. Murdoch, A. R. Rundell, J. Neal, and J. Summers (Hon. Secretaries). The various Standing Committees having been formed, and Governors to Participating Institutions for 1913 elected, it was decided to fix "Hospital Saturday" (Annual Special Collection) for October 11.

